# Augmentation of Cationic Antimicrobial Peptide Production with Histone Deacetylase Inhibitors as a Novel Epigenetic Therapy for Bacterial Infections

**DOI:** 10.3390/antibiotics4010044

**Published:** 2015-01-12

**Authors:** Roshan D. Yedery, Ann E. Jerse

**Affiliations:** Department of Microbiology and Immunology, F. Edward Hébert School of Medicine, Uniformed Services University of Health Sciences, 4301 Jones Bridge Road, Bethesda, MD 20814-4799, USA; E-Mail: ann.jerse1@usuhs.edu

**Keywords:** chromatin remodeling, histone deacetylase inhibitors, antimicrobial peptides, bacterial infections, anti-infectives

## Abstract

The emergence of antibiotic resistance seriously threatens our ability to treat many common and medically important bacterial infections. Novel therapeutics are needed that can be used alone or in conjunction with antibiotics. Cationic antimicrobial peptides (CAMPs) are important effectors of the host innate defense that exhibit broad-spectrum activity against a wide range of microorganisms. CAMPs are carried within phagocytic granules and are constitutively or inducibly expressed by multiple cell types, including epithelial cells. The role of histone modification enzymes, specifically the histone deacetylases (HDAC), in down-regulating the transcription of CAMP-encoding genes is increasingly appreciated as is the capacity of HDAC inhibitors (HDACi) to block the action of HDACs to increase CAMP expression. The use of synthetic and natural HDACi molecules to increase CAMPs on mucosal surfaces, therefore, has potential therapeutic applications. Here, we review host and pathogen regulation of CAMP expression through the induction of HDACs and assess the therapeutic potential of natural and synthetic HDACi based on evidence from tissue culture systems, animal models, and clinical trials.

## 1. Antimicrobial Peptides—An Innate Defense Against Microbial Pathogens

Confidence in currently licensed antibiotics to effectively treat and control bacterial infections has seriously waned in recent years with the emergence of multidrug resistance in several medically important bacterial species [1]. While new compounds are under development, novel strategies are needed to out-pace the selection for resistance mutations [2]. CAMPs are relatively small (<10 kDa), cationic and amphipathic peptides that form an important component of the host innate defense against invading pathogens [3]. CAMPs have been isolated from a wide variety of animals, both vertebrates and invertebrates, plants, fungi, and bacteria, and these innate effectors exhibit broad-spectrum activity against a wide range of microorganisms including Gram-positive and Gram-negative bacteria, protozoa, yeast, fungi and viruses [4]. CAMPs are broadly classified into five major groups based on three dimensional structural studies and amino acid composition, namely (a) peptides that form α-helical structures; (b) peptides rich in cysteine residues; (c) peptides that form β-sheets (d) peptides rich in specific amino acids e.g., histatin (rich in histidine), cathelicidins (rich in proline) and indolicidins (rich in tryptophan); and (e) peptides composed of rare and modified amino acids.

The mechanism by which CAMPs exert their antimicrobial activity involves disruption of the plasma membrane leading to the lysis of the target cell [5]. Hence, CAMPs are excellent candidate antimicrobial agents that can act against a broad range of pathogens alone or potentially, as adjunctive therapies for existing antibiotics. A few peptides have already entered clinical trials for the treatment of impetigo, diabetic foot ulcers and gastric helicobacter infections [6]. The potential therapeutic effect of CAMPs against sexually transmitted infections, including human immunodeficiency virus (HIV) and herpes simplex virus (HSV) infections [7], has also been investigated.

## 2. Pathogens Can Regulate HDAC-Mediated Expression of CAMPs

An alternative approach to directly challenging infectious agents with CAMPs is to induce CAMP expression therapeutically. Indeed, pathogens have evolved the opposite strategy of down-regulating CAMP expression to better establish themselves in the host. For example, several bacterial pathogens down-regulate the cathelicidin LL-37, secretory leukocyte protease inhibitor (SLPI), and, or human beta defensins (e.g., HBD-1, HBD-2, HBD-3) in tissue culture cells [8,9], and animal infection models [10]. These results are consistent with the detection of significantly lower concentrations of CAMPs in infected individuals. For example, significantly lower vaginal concentrations of SLPI were detected in non-pregnant women with gonorrhea or *Chlamydia* infections compared to uninfected healthy controls [11]. Similarly, down-regulation of LL-37 and HBD-1 transcription was detected in gut biopsies from individuals with *Shigella dysenteriae* [12], genetic evidence suggests pathogen-mediated suppression of gene transcription is responsible.

**Figure 1:**
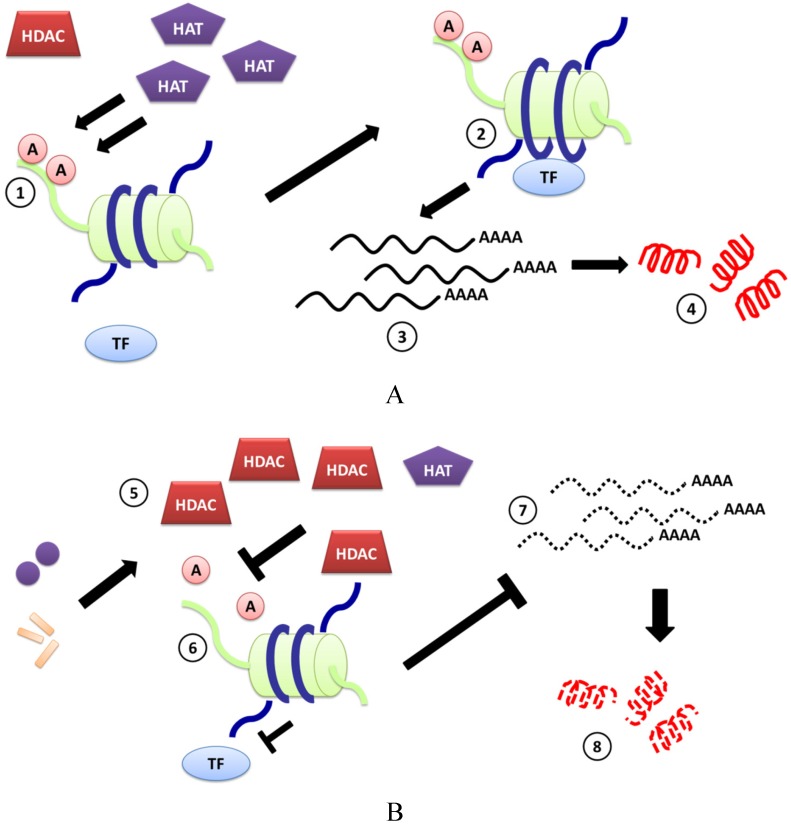

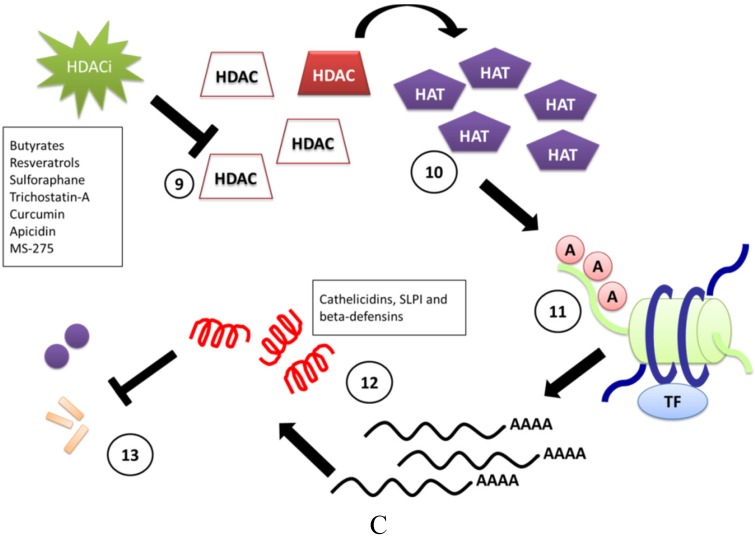
Pictorial description of chromatin remodeling resulting from the interplay between chromatin modifying enzymes and pathogens. (**A**) There is scientific evidence to assume that the enzyme histone acetylase adds acetyl groups to specific amino acids on the free N-terminal of histone proteins (not embedded in the octamer) (1), as a result of which the chromatin coiled around the histone octamers undergoes changes in spatial configuration exposing parts of DNA to which transcription factors bind (2). This change in DNA topography allows for a particular region of DNA to be transcribed and translated (3,4), thus regulating some important cellular functions including immune responses to pathogen invasion; (**B**) Several pathogens have evolved mechanisms to induce HDAC expression, which causes removal of acetyl groups attached to N-terminal histones, affecting the transcription and translation of many genes, including those involved in pathogen recognition, immunity and CAMP production (6–8). Examples include *Shigella dysenteriae*, *Vibrio cholerae* and *Anaplasma phagocytophilum* and *Porphyromonas gingivalis*. The mechanism(s) by which bacteria induce HDAC expression has not been well studied, although bacterial toxins or other cellular components (*i.e*., lipopolysaccharide, LPS) have been shown to play a role in some cases (5); (**C**) Chromatin remodeling can be regulated by HDACi, which directly interact with the HDAC enzyme and cause a state of hyperacetylation (9,10). This hyperacetylation can lead to a global change in gene expression. HDACi-induced over-expression of CAMP-encoding genes (11,12) has led to the idea of developing the HDACi as novel therapeutics for controlling bacterial infections in conjunction with antibiotic treatment (13).

The mechanism(s) by which bacteria down-regulate CAMP-encoding genes has not been resolved, but some bacterial pathogens can alter host gene expression at the level of chromatin remodeling. It is now well understood that regulation of gene expression can occur at several checkpoints: transcriptional, post-transcriptional, translational and post-translational stages. At the level of transcription, chromatin modifications play a very important regulatory role as chromatin remodeling is controlled by chromatin modifying enzymes [13], of which the histone deacetylases (HDAC) are an important family. HDAC control the availability of DNA binding sites to transcription factors by removing the acetyl groups from the surface of specific amino acids located in the N-terminal of histone proteins [14]. The balance between the histone acetylases (HA) and HDAC has been suggested to regulate transcription of several genes in multiple locations and collectively can cause global genomic and proteomic changes (Figure 1A).

The discovery that bacterial pathogens can alter host gene expression by altering the balance between HA and HDAC enzymes is a fascinating insight into the intimate evolution of microbes within a host. An early seminal study in 2009 by Garcia and colleagues [15] demonstrated that infection of THP-1 cells with *Anaplasma phagocytophilum*, the agent of human granulocytic anaplasmosis, led to suppression of a broad range of antimicrobial peptides and proteins namely cathelicidin, defensins (DEFB1, DEFB4, DEFA1, DEFA4 and DEFA6), azurocidin-1, lysozyme and cystatin A. A chromatin immunoprecipitation (CHIP) assay revealed a significant fold decrease in the acetylation and a proportional increase in the methylation of histone H3 at the promoters of the genes described above, suggesting the observed transcriptional changes were due to pathogen’s effects on chromatin remodeling enzymes, specifically histone deacetylases and methylases.

A handful of other reports have analyzed bacterial-induced regulation of HDAC expression. Yin and Chung reported that the oral pathogens *Poryphyromonas gingivalis* and *Fusobacterium nucleatum* modulated HDAC1 and, or HDAC2 expression in a human immortalized human keratinocyte cells and primary gingival epithelial cells [16]. *P gingivalis* caused the most significant suppression of HDAC gene transcription and lower numbers of bacteria able to significantly reduce HDAC-1 mRNA compared to HDAC-2 mRNA, which required a higher multiplicity of infection. It is not yet clear which HDACs influence the expression of specific CAMP-encoding genes. However, using small inhibitory RNAs, Kallsen and colleagues showed that knockdown of HDAC1, but not HDAC2 or HDAC3 expression in human lung adenocarcinoma A549 cells, increases HBD-1 gene expression, from which it is hypothesized that HDAC1 may play a regulatory role for HBD-1 expression in A549 cells [17]. The events by which other pathogens can suppress CAMP expression *in vitro* and *in vivo* are described in Figure 1B.

## 3. Induction of CAMPs by HDACi

HDAC inhibitors (HDACi) inhibit the activity of HDAC enzymes and several HDACi have been isolated from natural sources while others have been chemically synthesized [18]. HDACi can regulate transcription of a gene by inhibiting the HDAC enzyme from removing an acetyl group from histone tails. This inhibition leads to remodeling of the chromatin that is bound to histone octamers to increase the available binding sites on DNA for transcription factors and other regulatory proteins [19]. Recent studies using cDNA arrays have suggested that treatment of multiple cancer cell lines with HDACi affect gene expression in as many as 7%–10% of genes [20]. The number of genes affected by HDACi-treatment depended of several factors including time of culture, concentration, and the particular HDACi used [21].

HDAC inhibitors (HDACi) can prevent HDAC-mediated down-regulation of gene expression, and HDACi have been extensively evaluated for treating several cancers. A few HDACi have been approved for use by FDA or are in clinical trials [22]. Apart from their anti-cancer activities, HDACi have also been evaluated for other immunomodulatory properties and have attracted intellectual property interests from the pharmaceutical companies [23,24]. Interestingly, several reports in recent years have suggested that some HDACi induce the expression of CAMPs (Table 1). A review of the recent literature demonstrating CAMP-inducing activities of synthetic or natural HDACi follows (see Figure 1C).

**Table 1:** HDACi that have been evaluated for the capacity to induce CAMPs *in vitro* and *in vivo*.

HDAC Inhibitor	System Tested	Effect on CAMP mRNA Expression	Reference
Butyrate	Human lung epithelial cell line EBC-1	Cathelicidin ↑	[25]
	Human bronchial epithelial cell line VA10	Cathelicidin ↑ HBD-1 ↑	[26]
	Human airway epithelial cells NCI-H292	Cathelicidin ↑	[27]
	Human lung epithelial cell line A549	HBD-1 ↑	[17]
	Human primary gingival epithelial cells infected with *P. gingivalis* and *F. nucleatum*	HBD-2 ↑	[16]
	Human monocyte cell line U937	HBD-1 ↓	[26]
	Adult patients with shigellosis	Cathelicidin ↑	[28]
Resveratrol	Human keratinocyte cell line HaCaT	Cathelicidin ↑	[29]
	Topical administration in female hairless mice	Cathelicidin ↑	[30]
	Human monocyte cell line U937	Cathelicidin ↑	[29]
	Pseudomonas aeruginosa-infected A549 cells	HBD-2 ↓	[31]
Pterostilbene	Human monocyte cell line U937	Cathelicidin ↑	[29]
Polydatin	Human keratinocyte cell line HaCaT	HBD-2 ↑	[32]
Sulforaphane	Liver tissue from SFN-treated C57BL/6 mice	MBD-10 ↑	[33]
	Human intestinal epithelial cell lines Caco-2, HT-29 and SW480	HBD-2 ↑	[34]
	Mouse monocyte macrophage cell line RAW 264.7	SLPI ↑	[35]
	Nasal lavage from healthy human adults who ingested SFN-containing broccoli shake homogenate	SLPI ↑	[36]
Trichostatin-A	Human primary gingival epithelial cells infected with *P. gingivalis* and *F. nucleatum*	HBD-2 ↑	[16]
	Human lung epithelial cell lines A549 and NCI-H727	HBD-1 ↑	[17]
	Human airway epithelial cells NCI-H292	Cathelicidin ↑	[27]
Curcumin	Human cell lines: U937, HT-29 and HaCaT	Cathelicidin ↑	[37]
Apicidin	Human lung epithelial cell line A549	HBD-1 ↑	[17]
MS-275	Human lung epithelial cell line A549	HBD-1 ↑	[17]

## 4. Butyrates

Butyrate is a short chain fatty acid derived from the microbial fermentation of dietary fibers in the colon and are proven to have beneficial effects on health including the prevention of certain cancers, including colon cancer [38]. The beneficial effects of butyrate are attributed to its capacity to regulate gene expression through its action as a HDACi [39]. Ingram and colleagues were first to report that butyrate increased the level of acetylated histones in cultured HeLa and Friend erythroleukemic cells [40]. Butyrate inhibits most HDAC members except HDAC-6 and -10 (class III and class II HDACs, respectively). During inhibition of HDAC activity, HAT activity continues, which results in histone hyperacetylation [41]. Butyrate can exist in different forms and many forms have similar biological properties. These compounds have also have been studied for their ability to induce CAMPs. Butyrate was initially approved for treatment via an oral route for managing ulcerative colitis and urea cycle disorders [42,43]. However, due its foul smell, butyrate makes it unsuitable for oral therapy in humans. In recent literature, butyrate has been delivered to *in vivo* systems in the form of enema or creams to treat shigellosis and chronic dermatitis, respectively [28,44].

### 4.1. Butyrate-Induced Expression of Cathelicidins

Kida and colleagues were among the first to demonstrate the ability of sodium butyrate (SB) to induce cathelicidins in human lung epithelial cell line EBC-1 [25]. They observed a dose-dependent increase in the levels of cathelicidin mRNA expression in EBC-1 cells in response to SB-treatment, and results were confirmed at the protein level by immunoblot. Similar observations were made by Liu *et al.*, who reported reduced LL-37 expression in NCI-H292 human airway and nasal epithelial cells in response to SB-treatment [27]. Based on a luciferase reporter assay, the mechanism by which SB appears to increase LL-37 is through increased binding of transcription factor AP-1 to a specific region of the cathelicidin gene promoter sequence. CHIP assays suggested that SB-treatment of EBC-1 cells also augmented the acetylation of histone H3 and H4 at the cathelicidin gene promoter. This hypothesis was supported by immunoblot analysis, indicating that the augmentation of histone acetylation of the cathelicidin promoter participates in the SB-stimulated induction of cathelicidin gene expression in EBC-1 cells [25].

The therapeutic potential of SB as an anti-infective is further supported by a randomized, double-blind, placebo-controlled, clinical trial in adults with shigellosis. Delivery of SB (80 mM) as an enema to test subjects twice daily for 3 days resulted in higher LL-37 expression in the rectal epithelia compared to levels in subjects given saline in parallel. Stool concentrations of LL-37 remained significantly higher in the test group and an early reduction of macrophages, pus cells, IL-8 and IL-1β in the stool was observed as well as improvement in rectal histopathology [28].

Following the discovery of SB HDACi activity, Steinmann *et al.* [26] reported the ability of phenyl butyrate (PB) to induce cathelicidin expression in the immortalized human bronchial epithelial cell line, VA10. Incubation of VA10 with increasing doses of PB (0.25–4 mM) for 24 h resulted in a strong induction of cathelicidin mRNA [26]. Similar observations were also made with a human colonic adenocarcinoma HT-29 cells, human renal carcinoma A498 cells, and human leukemic monocyte lymphoma U937 cells, although the changes in LL-37 expression varied between cell types. Steinmann and colleagues also observed that co-stimulation of VA10 cells with 4 mM PB in the presence of vitamin D3 (1,25(OH)_2_D_3_) resulted in more than an 80-fold increase of cathelicidin mRNA levels over those treated with Vitamin D3 or PBA alone, and a ~100-fold increase compared to controls, indicating a synergistic induction [26]. These data were confirmed at the protein level with LL-37-specific antibodies. No significant change in histone acetylation on the proximal promoter of the cathelicidin gene was detected using a CHIP assay to assess the acetylation status of histones H3 and H4 in PB-treated VA10 cells, however, and thus in contrast to SB, the effect of PB on chromatin remodeling remains unknown.

### 4.2. Butyrate-Induced Expression of Beta-Defensins

Kallsen and colleagues observed that butyrate significantly upregulated the expression of HBD-1 in A549 cells in a temporal manner, with highest expression observed between 36 and 48 h post-treatment [17]. Yin and Chung subsequently reported that infection of gingival epithelial cells (GEC) with *P. gingivalis* caused a three-fold increase in HBD-2 expression and this effect was further enhanced when GECs were pretreated with 2 mM SB. A more pronounced increase in HBD-2 transcript levels was observed in *P. gingivalis*-infected GECs when pretreated with a combination of SB and Trichostatin-A (TSA) [16]. In contrast, *F. nucleatum* infection of GECs induced a strong response of HBD-2 gene expression, and while no additive effects were observed in cells pre-treated with SB, a combination of SB and TSA induced a very strong upregulation of HBD-2 mRNA [16]. Interestingly, the response to PB appears to be cell-specific. While PB treatment of VA10 cells significantly upregulated the transcription of the HBD-1 gene [26], PB treatment of U927 cells down-regulated DEFB1 gene expression. Based on this finding, the potential for cell-specific differences in responses to HDACi should be carefully considered when developing other HDACi for potential therapeutic use.

## 5. Resveratrol and Structurally-Related Molecules

Resveratrol (RESV; 3,5,4-trihydrostilbene) is a natural polyphenolic alcohol found in high quantities in plants and is produced in response to external stress, like UV irradiation, fungal infection or injury [45]. The molecule has attracted attention due to its beneficial properties in reducing the incidence of heart diseases. However, RESV also exhibits anti-oxidant, anti-inflammatory and anti-proliferative effects [46]. The compound has also been tested for its anticancer properties in tumors from different sites of the body [47].

RESV has been reported to demonstrate HDACi activity against specific members of class I and II HDAC enzymes. Molecular docking studies with HDAC-2, -4, -7 and -8 revealed that RESV fits into the binding pocket of all four HDACs and more specifically interacts with the binding pocket of the enzymes, which contains the active site with a zinc ion [48]. Further studies by Venturelli and colleagues employing specific fluorometric profiling assays revealed that treatment of HeLa cells with 50–100 µM RESV only moderately inhibited most members of HDAC family (HDAC 1–11). Significant inhibition of HDAC-1 and HDAC-4 was observed with a 100 µM dose of RESV.

In a recent study by Park and colleagues, RESV-treatment of HaCaT keratinocyte cells significantly induced the production of LL-37 mRNA [30]. Western blotting and ELISA assays with whole cell lysates and culture supernatants from with resveratrol-treated HaCaT cells showed a similar increase in LL-37 peptide levels. This RESV-mediated induction was dependent on the ceramide signaling pathway. Hence, co-incubation of HaCaT cells with RESV and N-oleoylethanolamine (NOE), an inhibitor of ceramidase, the enzyme that converts ceramide to sphingosine, significantly inhibited RESV-induced LL-37 expression at both the mRNA and protein level [30]. Similar observations were made with dimethylsphingosine and SKI, which are inhibitors that block the conversion of sphingosine to sphingosine-1-phosphate, suggesting that RESV-induced expression of LL-37 was regulated by the ceramide metabolic pathway. This hypothesis is further supported by the demonstration that topical administration of RESV on murine epidermis led to increased expression of the murine homolog of LL-37, cathelicidin-related antimicrobial peptide (CRAMP), and that this result was dependent on the sphingosine-1-phosphate-induced signaling [30].

In a separate study, Guo *et al.* observed that exposure of U937 cells to RESV for 18 h induced significant expression of cathelicidin. However, a stronger induction was observed when RESV was combined with 1,25(OH)_2_D_3_ [29]. Analysis of RESV-treated U937 cells by flow cytometry using anti LL-37 antibodies confirmed the inductive effects of RESV alone and in combination with 1,25(OH)_2_D_3_. Interestingly HaCaT cells treated with RESV at 10 µM for 18 h did not induce cathelicidin expression. However, when combined with 1,25(OH)_2_D_3_, RESV caused a three-fold increase in LL-37 mRNA level.

The efficacy of RESV to induce host effectors during a bacterial infection was brought into question by Cerqueira and colleagues, who found a significant down-regulation of HBD-2 transcripts in *Pseudomonas aeruginosa*-infected A549 cells when the cells were pre-treated with RESV (100 µmol) [31]. Further studies with different cell lines and pathogens are needed to better define the therapeutic potential of RESV against bacterial infection.

### 5.1. Pterostilbene

Pterostilbene is a stilbenoid found in rich quantities in blueberries and grapes, and is chemically related to RESV. It is a type of phytoalexin, which are agents produced by plants to fight infections. Like RESV, pterostilbene exhibits anti-oxidant, anti-inflammatory and anticancer activities [49]. Chen and colleagues reported that treatment of RPMI8226 multiple myeloma cells and HEK 293 cells with 10 µM of pterostilbene strongly induced histone acetylation and specifically prevented HDAC1 digestion by thermolysin [50]. Currently only one report suggests that pterostilbene induces CAMPs *in vitro*. Guo and group reported that pterostilbene treatment of U937 cells induced significant expression of cathelicidin. This increase was enhanced when pterostilbene was combined with 1,25(OH)_2_D_3_, leading to a three-fold increase in expression of cathelicidin compared with 1,25(OH)_2_D_3_ alone. Results from a flow cytometry assay designed to detect LL-37 peptide confirm these results [29]. In the same study, co-treatment of U937 cells with pterostilbene with calcipitriene and paracalcitol (pharmaceutical analogs of 1,25(OH)_2_D_3_) led to a 3–20 -fold increase in expression of cathelicidin at mRNA level.

### 5.2. Polydatin

Polydatin, also known as piceid (resveratrol-3-O-β-mono-D-glucoside, polydatin), is the glycoside form of RESV and is found in very high concentration in the grape *Polygonum cuspidatum*. Polydatin has the glucoside group bonded in position C-3 and substitutes a hydroxyl group giving rise to conformational changes of the molecule leading to increase in its biological properties. Like RESV and pterostilbene, polydatin is known to regulate oxidative and inflammatory pathways [51]. Currently, no reports exist to suggest that polydatin might be able to inhibit HDAC enzymes; however, it is highly anticipated that polydatin would also exhibit HDACi activities due to its structural similarity with RESV. A study by Ravagnan and colleagues revealed that pretreatment of HaCat cells with polydatin, alone or in combination with RESV for 24 h, induced HBD-2 expression at the mRNA level. This observation was confirmed by an ELISA for HBD-2, where a combination of polydatin and RESV induced as high as 191 ng/mL HBD-2 peptide in the culture supernatants compared to 9 ng/mL in untreated control HaCaT cells [32].

## 6. Sulforaphane

Sulforaphane (SFN) is a natural isothiocyanate, first isolated from broccoli, and a potent inducer of phase 2 detoxification enzymes and an inhibitor of phase 1 enzymes that activate chemical carcinogens. SFN has been shown to induce apoptosis and prevent tumors in mouse models [18]. SFN is also known to inhibit the HDAC family of enzymes. An early investigation of the HDACi activity of SFN by Myzak and colleagues showed that cytoplasmic and nuclear extracts from human embryonic kidney 293 cells treated with SFN had diminished HDAC activity along with a concomitant increase in histone acetylation compared to untreated cells [52]. A subsequent study from the same group demonstrated that the treatment of human colon cancer cells HCT116 with 35 μM SFN caused a significant decrease in HDAC-2 and HDAC-3 protein levels [53].

SFN has also been assessed for its ability to induce CAMPs both *in vitro* and *in vivo*. Treatment of intestinal epithelial cell lines (Caco-2, HT-29 and SW480) with SFN-induced HBD-2 mRNA expression in a time- and dose-dependent manner and increased levels of HBD-2 peptide as measured by an ELISA [34]. Similarly, SFN treatment of RAW 274.7 cells, a mouse leukaemic monocyte macrophage cell line, resulted in increased SLPI transcription [35]. In a recent study, Meyer and colleagues reported that healthy nonsmoking adults who ingested SFN-containing broccoli shake homogenate for three consecutive days demonstrated a significant increase in SLPI levels in nasal lavages. These investigators also showed that SFN-induced SLPI expression appears to be regulated by the Nrf2 transcription factor in that SLPI secretion was significantly decreased in cells transduced with Nrf2-specific shRNA [36]. This finding is consistent with an earlier study in which administration of SFN to C57BL/6J wild type and Nrf2 knockout mice resulted in increased expression of the beta defensin-10 gene [33].

Investigation of the use of SFN to treat *Helicobacter pylori* infections revealed other possible mechanisms by which SFN may have a therapeutic effect. SFN directly inhibited extracellular, intracellular, and antibiotic-resistant strains of *Helicobacter pylori* and prevented benzo[a]pyrene-induced stomach tumors in a mouse model when given orally [54]. The urease enzyme produced by *H. pylori* is critical for establishment of gastric colonization, and a recent follow-up study by the same group demonstrated that SFN inactivates *H. pylori* urease by forming a dithiocarbamate complex between the isothiocyanate group of SFN and cysteine thiols of urease [55]. Yanaka and colleagues also showed that *H. pylori*-infected mice given SFN-rich broccoli sprouts and a high-salt (7.5% NaCl) diet had reduced bacterial colonization, attenuated mucosal expression of TNF-α- and IL-1β, less corpus inflammation, and that high salt-induced gastric corpus atrophy was prevented [56]. The contribution of gastric cathelicidins or defensins were not evaluated in these study, but cannot be ruled out as a mechanism by which *H. pylori* colonization and inflammation were reduced.

The possibility that SFN may be effective against sexually transmitted infections was recently investigated by our group using *Neisseria gonorrhoeae* as the model pathogen, which is a pyogenic pathogen of the urogenital tract. We found that SFN-treatment of human endocervical carcinoma ME-180 cells led to an upregulation of HBD-2, HD-5 and SLPI gene expression [57] and that oral administration of SFN significantly reduced experimental colonization of female mice and suppressed the pro-inflammatory cytokine and chemokine response to infection (Yedery *et al.*, in preparation). These data support the potential use of HDACi alone or in conjunction with antibiotics to treat *N. gonorrhoeae*, which recently reached super-bug status due to the emergence of resistance to the extended cephalosporins, the last remaining monotherapy for empirical treatment of gonorrhea [58].

## 7. Trichostatin A

Trichostatin A (TSA, 7-[4-(dimethylamino)phenyl]-N-hydroxy-4,6-dimethyl-7-oxo-(2*E*,4*E*,6*R*)-2,4-heptadienamide), is a natural HDACi produced by two species of *Streptomyces*, *S. platensis* and *S. sioyaensis* [59]. The R-isomer of TSA was one of the first HDACi shown to increase the levels of histone acetylation in various mammalian cell lines [60]. TSA has also been well studied for its anti-inflammatory and anti-tumor activities.

TSA also induces CAMP expression in epithelial cells. TSA treatment of the lung epithelial cell lines A549 and NCI-H727 increased HBD-1 transcription, and a CHIP analysis with an anti-acetyl-histone H3 antibody revealed that TSA induced a 2.8-fold increase in histone H3 acetylation at the DEFB1 promoter [17]. Recently, Liu and colleagues reported that treatment of NCI-H292 human airway and human nasal epithelial cells with TSA increased the level of LL-37 transcription. Co-treatment with poly(I:C) did not affect the expression of the LL-37 gene, although poly(I:C) by itself weakly induced LL-37 expression [27]. A similar observation was made at protein level using an immunoblot assay. Importantly, studies Yin and Chung reported increased levels of HBD-2 mRNA when TSA-pretreated GECs were infected with *P. gingivalis* and *F. nucleatum* compared to uninfected controls [16]. This promising result suggests TSA may be effective against bacterial pathogens that are known to induce host HDACs and down-regulate CAMP expression.

## 8. Curcumin

Curcumin (CMN), or diferuloylmethane (1,7-bis-4(hydroxy-3-methoxyphenyl)-1,6-heptadiene-3,5-dione), is present within the rhizome of the plant *Curcuma longa*. CMN has generated significant interest due to its anti-cancer functions, which are attributed to its ability to inhibit specific molecular signaling pathways involved in carcinogenesis [61]. CMN also possesses anti-oxidant, anti-inflammatory, anti-proliferative and anti-angiogenic properties against several cancer cell types [62]. Studies have also demonstrated that CMN inhibits class I HDAC enzymes (HDAC 1-3 and HDAC-8), and Chen *et al.* reported that CMN is able to regulate cell proliferation and apoptosis in Raji cells by downregulating the expression levels of HDAC-1,HDAC-3 and HDAC-8 proteins and by upregulating acetylated histone H4 protein expression [63].

CMN treatment of tissue culture cells also results in increased CAMP expression. In a recent report, Guo and group demonstrated that CMN treatment of U937 and HT-29 cells significantly induced expression of LL-37, but not in HaCaT cells. Elevated intracellular LL-37 levels were also detected in CMN-treated U937 cells as measured by intracellular staining and FACS [37]. Using a luciferase reporter assay it was observed that induction of cathelicidin by CMN does not require Vitamin D response elements (VDRE). Further, a CHIP assay with CMN-treated U937 cells demonstrated that CMN did not increase VDR binding to cathelicidin promoter suggesting that CMN-induced human LL-37 expression occurs through a VDR- independent mechanism [37].

## 9. Apicidin

Apicidin (APD) [cyclo(NO-methyl-L-tryptophanyl-L-isoleucinyl-D-pipecolinyl-L-2-amino-8-xodecanoyl)], is a fungal metabolite that was originally known for its broad spectrum antiprotozoal activity against *Apicomplexan* parasites and *Plasmodium berghei in vitro* [64]. Later studies demonstrated the ability of APD to inhibit mammalian HDAC. The ability of APD to interact with HDAC depends on its unique structure, which contains an ethyl ketone as a potential zinc-binding group, a long alkyl chain as a linker, and a cyclic tetrapeptide [65]. A structural derivative of API, API-D, exhibits selective inhibition of class I subtypes HDAC-1, HDAC-2 and HDAC-3 [66]. Recent studies showed that APD induced expression of HBD-1 in A549 cells in a time-dependent fashion with mRNA levels achieving a seven-fold increase compared to untreated controls at 36 h post treatment.

## 10. MS-275

MS-275, also known as entinostat, is an HDACi that belongs to the 2-aminophenyl benzamides. MS-275 inhibits class I HDAC enzymes, with a high affinity for HDAC-1, HDAC-2 and HDAC-3, but has a relatively weak affinity for HDAC-8 [67]. Recently Kallsen et.al, reported that MS-275-treatment of A549 lung epithelial cells exhibited a temporally significant increase in the expression of HBD-1 mRNA. Results from CHIP assays revealed that acetylation of histone H3 and the trimethylation of lysine 4 at histone 3 (H3K4), which are histone modifications associated with transcriptionally active chromatin, were increased at the DEFB-1 promoter after treatment with MS-275 for 36 h [17].

## 11. Anti-Inflammatory Properties of HDACi

HDACi have also been extensively studied for their ability to control and regulate inflammation triggered by microbial ligands. Early studies by Segain and colleagues demonstrated that butyrate suppressed lipopolysaccharide (LPS)-induced secretion of cytokines by peripheral blood mononuclear cells. The suggested mechanism was that butyrate prevented the transmigration of NF-kB from the cytoplasm to the nucleus [68]. Tedelind *et al.* demonstrated that butyrate and propionate reduce inflammation-mediated tissue insult in a mouse colitis model [69]. In another report, Zhing and group reported the anti-inflammatory activity of RESV in LPS-exposed microglial cells. Their studies revealed a RESV-mediated down-regulation in phosphorylation levels of the transcription factors: NF-κB, CREB and MAPKs family in an mTOR-dependent manner [70]. Meng and colleagues observed that curcumin inhibited LPS-induced inflammation in rat vascular smooth muscle cells. The mechanism was dependent on the inhibition of the TLR4-MAPK/NF-κB pathways via blockage of NADPH-mediated intracellular ROS production [71]. Recently Brandenburg *et al.* demonstrated the ability of SFN to attenuate LPS-induced IL-1β, IL-6 and TNF-α expression in microglia; these investigators also reported that SFN significantly decreases the LPS-induced nitric oxide in a concentration-dependent manner [72]. In summary, these studies suggest that HDACi may have the therapeutic benefit of effectively regulating microbial infections due to the dual capacity of both inducing CAMPs and inhibiting the harmful effects of pathogen-mediated inflammation and tissue damage.

## 12. Conclusions and Future Directions

Due to increasing antimicrobial resistance and non-availability of vaccines for many pathogens, there is an urgent need to identify novel therapeutic strategies to combat the spread of these infections. The studies and observations reviewed in this article suggest that HDACi could serve as novel candidates, either as new antibiotics or adjunctive therapies in combination with existing control measures. Further investigation is needed, however; in particular, the testing of HDACi in infection models is needed to determine whether HDACi can out-compete the capacity of some bacterial pathogens to down-regulate CAMP expression. It is also important to determine whether HDACi can increase CAMP concentrations at mucosal surfaces to a level that overcomes other bacterial defenses against CAMPs, such as surface modifications that reduce CAMP binding and active efflux of internalized peptides. More experimentation is also needed to better understand the mode of action of the different HDACi identified to date. HDACi targeting specific HDAC enzymes will have an advantage over pan-inhibitors which inhibit multiple HDAC members. Also, profiling HDACi for their ability to induce individual CAMPs may generate more candidates for further evaluation with animal models and clinical trials. Subject to available technology, progress in this area is attainable, and should be supported due to the pressing need for novel anti-infectives.
